# Unrevealing Reliable Cortical Parcellation of Individual Brains Using Resting-State Functional Magnetic Resonance Imaging and Masked Graph Convolutions

**DOI:** 10.3389/fnins.2022.838347

**Published:** 2022-03-09

**Authors:** Wenyuan Qiu, Liang Ma, Tianzi Jiang, Yu Zhang

**Affiliations:** ^1^Research Center for Healthcare Data Science, Zhejiang Lab, Hangzhou, China; ^2^Brainnetome Center, Institute of Automation, Chinese Academy of Sciences, Beijing, China; ^3^National Laboratory of Pattern Recognition, Institute of Automation, Chinese Academy of Sciences, Beijing, China

**Keywords:** functional connectivity, cortical parcellation, intersubject variability, topographic variability, resting-state fMRI (rfMRI), test–retest reliability, graph neural network

## Abstract

Brain parcellation helps to understand the structural and functional organization of the cerebral cortex. Resting-state functional magnetic resonance imaging (fMRI) and connectivity analysis provide useful information to delineate individual brain parcels *in vivo*. We proposed an individualized cortical parcellation based on graph neural networks (GNN) to learn the reliable functional characteristics of each brain parcel on a large fMRI dataset and to infer the areal probability of each vertex on unseen subjects. A subject-specific confidence mask was implemented in the GNN model to account for the tradeoff between the topographic alignment across subjects and functional homogeneity of brain parcels on individual brains. The individualized brain parcellation achieved better functional homogeneity at rest and during cognitive tasks compared with the group-registered atlas (*p*-values < 0.05). In addition, highly reliable and replicable parcellation maps were generated on multiple sessions of the same subject (intrasubject similarity = 0.89), while notable variations in the topographic organization were captured across subjects (intersubject similarity = 0.81). Moreover, the intersubject variability of brain parcellation indicated large variations in the association cortices while keeping a stable parcellation on the primary cortex. Such topographic variability was strongly associated with the functional connectivity variability, significantly predicted cognitive behaviors, and generally followed the myelination, cytoarchitecture, and functional organization of the human brain. This study provides new avenues to the precise individualized mapping of the cortical areas through deep learning and shows high potentials in the personalized localization diagnosis and treatment of neurological disorders.

## Introduction

Brain atlas has been an important tool to understand the neural basis of human cognition and to study the functional organization of the human brain (Ungerleider and Desimone, 1986; Felleman and Van Essen, 1991; Amunts and Zilles, 2015). Neuroanatomists have built a variety of brain atlases to depict cyto-, myelo-, and receptor architectures using postmortem brains (Brodmann, 1909; Zilles and Amunts, 2010; Amunts et al., 2020). Recent advances in noninvasive neuroimaging techniques, such as functional magnetic resonance imaging (fMRI), provide an opportunity to explore the functional organization of the living human brain. There has been a rich and fast-growing literature on the functional brain parcellation using either spontaneous low-frequency fluctuations of fMRI activity or the aggregation of activation maps across different cognitive tasks (Blumensath et al., 2013; Eickhoff et al., 2015; Eickhoff et al., 2018a,b). The majority of current approaches focused on the group representative functional mapping of the cerebral cortex, which may provide useful insights into the intrinsic organizational principles of the human brain (Buckner et al., 2013; Wig, 2017), but ignore the variability of individual brains in areal size, location, spatial arrangement, and connectivity patterns (Mueller et al., 2013; Zuo and Xing, 2014). The precise mapping of individualized functional areas is a critical step toward better understanding of the structural–functional relationship of the human brain that underlying cognition and behavior (Wang et al., 2015; Kong et al., 2019, 2021) as well as for personalized localization diagnosis and treatment of neurological disorders (Mueller et al., 2015; Wang et al., 2020).

Traditional individualized mapping of brain atlas has relied on the linear and nonlinear registration based on the structural images in the volume space or cortical surfaces. Modern machine learning algorithms provide analytic tools to align cortical areas using neuroimaging data across multiple modalities, including myelin maps and functional localizers (Robinson et al., 2018), as well as anatomical (Ma et al., 2021) and functional connectivity fingerprints (Wang et al., 2015). As one of the most commonly used features for individualized brain mapping, functional connectivity has been shown to reveal individual-specific topographic organization that better predicted cognitive functions and behaviors (Wang et al., 2015; Cui et al., 2019; Kong et al., 2019; Li et al., 2019). However, the reliability and validity of such topographic variability has been one major concern considering that the fMRI signals are highly contaminated by noises of various physiological processes and head motions. By explicitly separating actual intersubject variability from noise components (evaluated by multiple sessions of the same subject), studies have shown that the individualized parcellation not only exhibited better functional homogeneity at rest and during cognitive tasks (Kong et al., 2021), but also captured reliable and inheritable variability in the topographic organization of the human brain (Anderson et al., 2021), demonstrated by the genetic effects of topographic variability. Yet, this multisession hierarchical Bayesian model (MS-HBM) used a global concentration parameter to model the heterogeneity of functional connectivity for different brain parcels, and resulted in similar levels of topographic variability and heritability among the primary and association cortices by treating each area equally, which is in congruence with the well-known sensory-fugal gradient in the myelination, cytoarchitecture, and functional organization of human brain.

In this study, we proposed a masked graph neural network (GNN) architecture to learn the reliable functional characteristics of each brain parcel using fMRI data from a large population and to apply such information to infer the areal probability of each vertex on unseen subjects. Specifically, we constructed a vertex-level brain graph for each subject and embed the whole-brain functional connectivity profiles as signals on the graph. Then we used high-order graph convolution to integrate the local connectivity context of each vertex such that the fluctuations in functional connectivity profiles were evaluated among a small neighboring area in the cortical surface rather than on each vertex individually, largely suppressing the noise effects from individual fMRI runs. Besides, we trained hundreds of graph convolutional kernels at each graph convolution layer to encode the variational organizational principles among cortical areas. Moreover, we implemented subject-specific confidence masks in the GNN model to maintain a consistent global topographic organization among subjects while preserving the intersubject variability in brain parcellation especially for vertices around the areal borders. Compared with the baseline approaches including the group-registered atlas and MS-HBM, our model generated highly consistent and replicable parcellation maps on individual brains, along with better functional homogeneity at rest and during cognitive tasks. Moreover, the topographic variability generally followed a sensory-fugal gradient from primary and unimodal areas to heteromodal areas, with high variations in the association cortices while keeping a stable parcellation on the primary cortex. More importantly, the topographic variability was strongly associated with individual variability in functional connectivity profiles and cognitive behaviors.

## Materials and Methods

### Data Acquisition and Preprocessing

We used two independent datasets acquired from the Human Connectome Project (HCP) dataset, consisting of T1-weighted (T1w) data, resting-state functional MRI (rs-fMRI), as well as task-fMRI data for each subject. The individualized parcellation model was first trained and evaluated on a large dataset consisting of 1,022 subjects acquired from the Human Connectome Project S1200 release^1^. We then evaluated the test–retest reliability of the model on the second dataset, consisting of 44 subjects acquired from the HCP test–retest datasets. Whole-brain echo-planar imaging (EPI) acquisitions were acquired with a 32-channel head coil on a modified 3T Siemens Skyra with TR = 720 ms, 2.0-mm isotropic voxels, using a multiband sequence. Each subject underwent two fMRI sessions on separate days, including two runs of 14-min resting-state and seven task fMRI scans (we only used fMRI data with the left to right (LR) phase encoding in the current study). The task-fMRI database includes seven cognitive domains, which are emotion, gambling, language, motor, relational, social, and working memory. The detailed description of data collection and task paradigms can be found in Barch et al. (2013).

We used the minimal preprocessed fMRI data from the HCP pipelines^2^ : (1) fMRIVolume pipeline generates “minimally preprocessed” 4D time series (i.e., “.nii.gz” file) that includes gradient unwarping, motion correction, fieldmap-based EPI distortion correction, brain-boundary-based registration of EPI to structural T1-weighted scan, nonlinear (FNIRT) registration into MNI152 space, and grand-mean intensity normalization. (2) fMRISurface pipeline projects fMRI data from the cortical gray matter ribbon onto the individual brain surface (fs_LR32K surface space) and then onto template surface meshes (i.e., “dtseries.nii” file), followed by surface-based smoothing using a geodesic Gaussian algorithm. Additional preprocessing steps were applied on rs-fMRI data before the calculation of functional connectivity, including regressing out the signals from white matter and csf, and the bandpass temporal filtering on frequencies between 0.01 and 0.1 Hz. Further details on fMRI data acquisition and preprocessing can be found in Glasser et al. (2013) and Barch et al. (2013).

### Construction of Individual Brain Graph

The preprocessed fMRI data were mapped onto the standard surface template with 32k vertices per hemisphere. After removing confounding vertices on the medial surfaces, the cortical mask consists of 59,412 cortical vertices. Then an adjacency matrix A was generated from the surface mesh files, with A_ij_ = 1 indicating that the two vertices i and j are shared in a common triangle in the cortical mesh. Since all subjects have already been registered onto the standard surface template during data preprocessing, the adjacency matrix A was also shared across all subjects. As such, a binary brain graph 𝒢 = (𝒱,ℰ) was constructed for each individual brain, with the node 𝒱 indicating each vertex in the cortical mesh, and the edge ℰ specified by the adjacency matrix indicating whether two nodes are connected or not. The resulting brain graph is sparsely connected and highly localized in space, with each vertex only connecting with two to six nearest vertices on average.

### Functional Connectivity Profile as Graph Signals

For each vertex in the cortical mask, we calculated its functional connectivity profile by calculating Pearson correlations on preprocessed fMRI signals and treated it as a feature vector on each node of the graph. In order to save the computational cost and complexity, we did not use the vertex-wise functional connectivity maps but instead calculated the connectivity fingerprints evaluated on hundreds of functional region of interest (ROIs) from a group atlas, e.g., Schaefer400 (Schaefer et al., 2018). The connectivity fingerprint of each cortical vertex *x* represents the probability of assigning the seed vertex to the same label of each functional ROI. These connectivity profiles were then concatenated and embedded in the individual brain graph as graph *X* ∈ ℝ^*N* = *F*^, where *N* indicates the number of cortical vertices, and *F* indicates the number of features in the connectivity profiles.

### ChebNet Convolution on the Brain Graph

After defining the graph 𝒢 = (𝒱,ℰ) with signals *X* ∈ ℝ^*N* = *F*^ for each subject, a GNN architecture was applied on the combined graph data $\tilde{𝒢}=\left(𝒱,ℰ,X\right)$ with the aim of integrating the context information of functional connectivity profiles at each vertex from its spatial neighbors by using graph convolutions. Graph convolution relies on the graph Laplacian, which is a smoothing operator characterizing the magnitude of signal changes between adjacent nodes. The normalized graph Laplacian is defined as:


(1) $$L=I-D^{-1/2}\,A\,D^{-1/2}$$

where *D* is a diagonal matrix of node degrees, *I* is the identity matrix, and *A* is the adjacent matrix of the graph. The eigen decomposition of Laplacian matrix is defined as *L* = U△U*^T^*, where U = (*u*_0_,*u*_1_,⋯*u*_*N*−1_) is the matrix of Laplacian eigenvectors and is also called graph Fourier modes, and △ = diag(λ_0_,λ_1_,⋯λ_*N*−1_) is a diagonal matrix of eigenvalues, specifying the frequency of the graph modes. The convolution between the graph signals *X* ∈ ℝ^*N* = *F*^ and a graph filter *g*_θ_ ∈ ℝ^*N* = *F*^ based on graph 𝒢, is defined as their element-wise Hadamard product in the spectral domain:


(2) $$x{*}_{𝒢}g_{\theta }=U\,\left(U^{T}\,g_{\theta }\right)⊙\left(U^{T}\,x\right)=U\,G_{\theta }\,U^{T}\,x$$

where *G*_θ_ = *diag*(*U^T^g*_θ_)and θindicate a parametric model for graph convolution *g*_θ_, *U^T^x* projects the graph signals onto the full spectrum of graph modes. To avoid calculating the spectral decomposition of the graph Laplacian, ChebNet convolution (Defferrard et al., 2016) uses a truncated expansion of the Chebyshev polynomials, which are defined recursively by:


(3) $${\text{T}}_{k}\,\left(x\right)=2\,x\,{\text{T}}_{k-1}\,\left(x\right)-{\text{T}}_{k-2}\,\left(x\right),{\text{T}}_{0}\,\left(x\right)=1,{\text{T}}_{1}\,\left(x\right)=x$$

Consequently, the ChebNet graph convolution is defined as:


(4) $$x{*}_{𝒢}g_{\theta }=\sum_{k=0}^{K}\theta_{k}\,{\text{T}}_{k}\,\left(\tilde{L}\right)\,x$$

where $\tilde{L}=2\,L/\lambda_{m\,a\,x}-I$ is a normalized version of graph Laplacian with λ_*max*_ being the largest eigenvalue, θ_*k*_ is the model parameter to be learned at each order of the Chebychev polynomials. It has been proven that the ChebNet graph convolution was naturally *K*-localized in space by taking up to *K*th order Chebyshev polynomials (Defferrard et al., 2016), which means that each ChebNet convolutional layer integrates the context of brain activity within a *K*-step neighborhood. We found that high-order graph convolutions might introduce over smoothing issues and result in decreased functional homogeneity in fMRI signals (Supplementary Figure 3). In this study, we used the third-order graph convolutions in our GNN architecture.

### Masked Semi-Supervised Graph Convolutional Neural Network for Individualized Cortical Parcellation

The GNN model takes the constructed brain graph $\tilde{𝒢}=\left(𝒱,ℰ,X\right)$ as inputs, where 𝒱 is the set of 32k vertices in the cortical surface, ℰ is the set of edges indicating whether two vertices share a common triangle in the surface, and *X* ∈ ℝ^*N = F*^ is the set of feature vectors indicating the functional connectivity profiles of each vertex. A series of third-order graph convolution were then applied on the graph signals, with 64 kernels in the first graph convolutional layer and 201 kernels in the second layer. The learned graph representations of the last layer were transformed to a 201-dimensional probability vector using the SoftMax function. The loss function of the proposed model was defined as follows:


(5) $$L\,o\,s\,s=\sum_{n}\sum_{v}w_{n,v}\,\sum_{k}y_{n,v,k}\,\log\,\left({\text{p}}_{v,k}\right)$$

We used the K–L divergence to compute the difference between the group prior *y*_n,v,k_ and the predicted probability *p*_v,k_ at each vertex *v* for each region *k*. The weight of uncertainty *w*_n,v_ was evaluated for each vertex, inferred by a subject-specific confidence mask. The confidence mask was generated as follows (Supplementary Figure 1): (1) initial parcellation: assigning each vertex in the cortical mask to the corresponding parcel with the highest similarity in functional connectivity profiles; (2) group-level alignment: excluding vertices in the initial parcellation with different parcel labels as the group atlas; (3) subject-level alignment: overlapping parcellation maps across all available sessions of a single subject. The resulting confidence map contains around half of the cortical mask (56% of vertices) that contributed to the final loss function in all subjects. The benefits of using the above loss function include: (1) high contributions from the vertices near the center of brain parcels, preserving a consistent global topographic organization across subjects; and (2) small contributions from the vertices around the areal borders, retaining the intersubject variability to some degree by introducing mismatching labels across subjects.

The proposed pipeline of individualized brain parcellation (as shown in Supplementary Figure 5) was trained on 50 subjects with two sessions for 100 epochs with the batch size set to 1 (processing one subject at a time), evaluated on all other subjects in the HCP S1200 dataset as well as the test–retest dataset. To avoid overfitting, an early stopping of 10 epochs was used, along with an additional l_2_ regularizations of 0.0005 on model weights and a dropout rate of 0.5 on each graph convolutional layer. The best model over 100 training epochs was saved and further evaluated on the independent test sets from HCP S1200 and test–retest datasets.

### Comparison With Alternative Machine Learning Approaches

Many approaches of individualized brain parcellation have been proposed in the literature, for instance, by using an iterative clustering of fMRI signals (Wang et al., 2015) or hierarchical inference through a multilevel Bayesian model (Kong et al., 2021). We included two individualized methods as baseline approaches in the current study. The first approach aligned the group atlas into individual brains using the multimodal alignment protocol (Robinson et al., 2018), which utilized myelin maps, resting state network maps, and visuotopic maps to align cortical areas. The second approach modified the individual mapping of brain atlas using a multisession hierarchical Bayesian model (Kong et al., 2019, 2021), by explicitly modeling the variability in functional connectivity at the levels of intra- and intersubject. The preprocessed HCP fMRI dataset has already included the copies of the first approach (i.e., the MSMALL version), for which we compared the functional homogeneity between our individualized brain parcellation and group-registered brain atlas (see section “Resting State Functional Homogeneity of Brain Parcels”). For fair comparisons with the second approach, we reran the MS-HBM approach on the same group of subjects along with the same preprocessing steps as in our model and compared the distribution of topographic variability between the two approaches (see section “The Intersubject Reliability and Variability of Individual Properties”). We chose the Schaefer400 atlas (Schaefer et al., 2018) as the referenced group atlas for all approaches and used the author-suggested model parameters for rerunning the MS-HBM approach (Kong et al., 2021), including priors of group spatial (100), markov random field (MRF) smoothing (50), and gradient-based spatial localization (50).

### The Intersubject Reliability and Variability of Individual Properties

The reliability of individual parcellation and its intersubject variability were evaluated on both HCP S1200 and test–retest datasets. Each subject underwent two (HCP S1200 dataset) or four (test–retest dataset) fMRI sessions. The reliability of individual parcellation was evaluated by the averaged Dice coefficients among all possible pairs. The Dice coefficient was first evaluated on each brain parcel using the equation (2 × A∩B)/(A + B), where A and B indicate two different parcellation schemes, and then averaged across the whole cortex. The effect size of the intersubject variability was measured by Cohen’s *d*, representing the standardized difference between the mean values of two distributions, defined as follows:


(6) $${\text{Cohen}}^{\prime }\,\text{s}\,d=\frac{\mu_{i\,n\,t\,e\,r}-\mu_{i\,n\,t\,r\,a}}{\sqrt{\sigma_{i\,n\,t\,e\,r}^{2}+\sigma_{i\,n\,t\,r\,a}^{2}}}$$

where μ_inter_ and σ_inter_ represent the mean and standard deviation of the variabilities between each pair of subjects, while μ_intra_ and σ_intra_ represent the mean and standard deviation of the variabilities between different fMRI scans of the same subject. We used different indices to measure the variability of parcellation maps and connectivity profiles. Specifically, the variability in brain parcellation (i.e., areal topographic variability) was measured by the Dice coefficient between two parcellation maps. The variability of connectivity profiles (i.e., functional connectivity variability) was measured by the correlation coefficients of the functional connectivity profiles between two fMRI runs.

For further validation of the biological basis of the intersubject variability in brain parcellation, additional association analyses were conducted for the areal topographic variability, including the variability of connectivity profiles, distribution of T1w/T2w myelin ratio, as well as the sensory-fugal map of laminar differentiation. As a quantitative measure of the myelin content of cerebral cortex, the myelin ratio map was defined as the ratio of T1w and T2w structural images on each subject and then averaged across all subjects on the HCP S1200 release (Glasser and Van Essen, 2011). The cortical myelin map was then mapped onto the chosen group atlas by averaging the T1w/T2w ratio within each ROI. The laminar differentiation map identifies four different cortical types based on their cytoarchitectonic organization, namely, paralimbic, heteromodal, unimodal, and idiotypic areas (Mesulam, 1998). A cortical mapping of laminar differentiation was generated by assigning each ROI in the chosen group atlas to one of the four types.

### Resting State Functional Homogeneity of Brain Parcels

Functional homogeneity in resting-state fMRI was defined as the averaged Pearson’s correlations of fMRI signals between all pairs of vertices within each parcel, adjusted for the parcel size (Kong et al., 2019, 2021). Higher functional homogeneity indicates similar brain dynamics of fMRI activity within the same parcel. The functional homogeneity was evaluated on the test set from the HCP S1200 (in a total of 928 subjects, after excluding the training data, subjects included in the test–retest data, and some abnormal data with missing functional scans) for both group-registered parcellation and masked semi-supervised graph convolutional neural network (MSGCN) individualized brain parcellation. A two-sided paired *t*-test was applied to test the significant differences of functional homogeneity between the two approaches.

### Regional Variability and Homogeneity in Task Activation

We chose one representative task contrast from each of the seven cognitive domains: right-hand movement for the motor task, two back conditions on tool images for working memory, math vs. story for language processing, faces vs. shapes for emotional processing, theory of mind vs. random motion for social cognition, reward for the gambling task, and relational processing vs. pattern match for relational processing. For each task contrast, we evaluated the regional variability and homogeneity in the task activations from the test subjects of the HCP S1200 dataset. Regional variability in task activation was evaluated by calculating the standard deviation of brain activation within each parcel by using the beta maps derived from the generalized linear model (GLM) analysis. Lower task variability indicates small variations of task activations within each brain parcel and large variations along the areal boundaries. Regional homogeneity in task activation was evaluated by the mean activation strength in each brain parcel. Higher task homogeneity indicates better functional alignment in the task activations across different subjects.

### Prediction Cognitive Behaviors Using Individualized Parcellation

To further validate that individualized parcellation improves the intersubject functional alignment in brain organization and captures meaningful aspects of human cognition, we performed another experiment to predict cognitive behaviors using the intersubject variability in brain parcellation. A kernel regression method was used to predict the behavioral score of the test subject based on the assumption that similar topography in brain parcellation induced similar performance in behavior, defined as follows:


$$y\approx \sum_{i}D\,i\,c\,e\,\left(l,l_{i}\right)\,y_{i}$$

where *y_i_* represents the behavioral score of the *i*-th subject in the training set, *l_i_* represents the parcellation map of the subject *i*, *Dice*(*l*,*l*_*i*_) represents the Dice coefficient of parcellation maps between the test subject and the subject *i*, and *y* represents the behavioral score of the test subject. An additional l_2_-regularization term was used to prevent overfitting issues, with the regularization parameter determined by a fivefold cross-validation procedure.

For each of the 58 cognitive behaviors, a prediction model was trained and evaluated on 928 individualized brain parcellations. Specifically, we trained the prediction model on 200 subjects and evaluated it on the rest of the 728 subjects. First, we used a five-fold cross-validation strategy to determine the optimal prediction model (including model parameters and the *l_2_*-regularization parameter). The model was then used to predict the behavioral scores on the test subjects. Finally, the performance of the prediction model was evaluated by calculating the Pearson correlation of predicted and measured behavioral scores.

## Results

### Masked Semi-Supervised Graph Convolutional Neural Network Model for Individualized Brain Parcellation

The MSGCN model (as shown in Supplementary Figure 5) was evaluated using 1,022 subjects from HCP S1200 dataset with two fMRI sessions for each subject, among which 40 subjects were randomly chosen for model training, 10 subjects for validation, and the rest of the datasets used for model testing. During model training, a vertex-level brain graph was first constructed from individual T1-weighted brain images, indicating the spatial adjacency between any two vertices in the cortical surface. The functional connectivity profiles of each vertex were then embedded in the brain graph as graph signals. Finally, the areal probability of each vertex was inferred by using a two-layer graph convolution architecture with a subject-specific confidence mask. As a result, the MSGCN model extracted reliable functional characteristics of each brain parcel inferred from a large population and generalized over data of unseen subjects by revealing reliable parcellation maps for each of the test subjects.

### Topographic Organization of Individual Parcellation and Its Test–Retest Reliability

**FIGURE 1:**
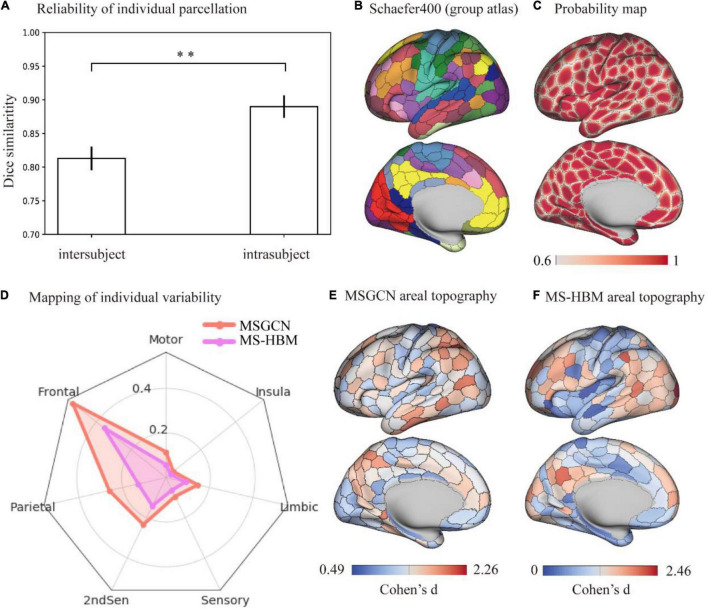
Topographic organization of individual parcellation and its reliability measured on the HCP S1200 dataset. **(A)** Reliability of individual parcellation. The masked semi-supervised graph convolutional neural network (MSGCN) individualized parcellation showed significantly higher similarity for intrasubject (0.889 ± 0.025) than intersubject (0.810 ± 0.021), detected by a paired *t*-test (*p* < 0.001). **(B)** Cortical mapping of the group atlas. We used a similar color scheme as the Yeo-7 networks for the Scaefer400 atlas, while the areal borders were delineated by the gray line. **(C)** Probability map of individualized parcellation maps. The parcellation maps on all subjects were summarized into a population probability map, indicating the probability of assigning the vertex to the same parcel among the HCP population. A population threshold of 60% was applied to the probability map. **(D)** Distribution of intersubject variability in brain parcellation among functional networks. We used Cohen’s *d* to evaluate the effect size of variability in individualized parcellation generated by MSGCN (orange line) and multisession hierarchical Bayesian model (MS-HBM) (purple line), both of which showed large variations in the association cortex including “Ffontal,” “parietal,” and “2ndSen” regions and low variability in the motor and sensory cortices. However, the MSGCN model detected higher variability in high-order cognitive areas, especially in the frontal and parietal regions. **(E)** Cortical mapping of intersubject variability for the MSGCN model. **(F)** Cortical mapping of intersubject variability for the MS-HBM. ^**^p-value < 0.01.

The cortical parcellation maps on individual brains followed a similar global topographic organization as the group atlas (e.g., Schafer400 atlas), indicating a relatively high similarity [dice = 0.847 ± 0.013 (mean ± std)] among all fMRI sessions of 1,022 HCP subjects. First, a stable brain parcellation scheme was revealed such that the majority of vertices showed consistent parcel labels across subjects, with over 75% of cortical vertices showing a high population probability >0.6. We observed near-perfect alignment at the center of brain parcels along with notable variability around the areal borders (Figure 1C). Second, the reliability of individual parcellation, measured by the intra- and intersubject similarity (Figure 1A), showed significantly higher consistency among multiple sessions of the same subject (dice = 0.889 ± 0.025) than between different subjects (dice = 0.810 ± 0.021), as revealed by a paired *t*-test (*p* < 0.001). Moreover, the intersubject variability in brain parcellation (i.e., areal topographic variability), evaluated by the Cohen’s *d* effect size among individualized parcellation maps, was not uniformly distributed in the cerebral cortex, but rather followed the functional organizational principles (Figure 1E). After mapping the areal topographic variability onto the seven functional networks (Ito, 2018), we found consistent parcellation schemes for the motor and sensory cortices (i.e., low variability) and divergent brain parcels for the association cortices (i.e., high variability) including frontal, parietal, and temporal areas (Figure 1D). Compared with another individualized parcellation approach, e.g., the MS-HBM, our MSGCN model revealed higher areal topographic variably, in general (Supplementary Figure 4), with a similar level of areal topographic variability in the primary cortex but detecting much higher variability in the secondary and association cortices (Figure 1D).

**FIGURE 2:**
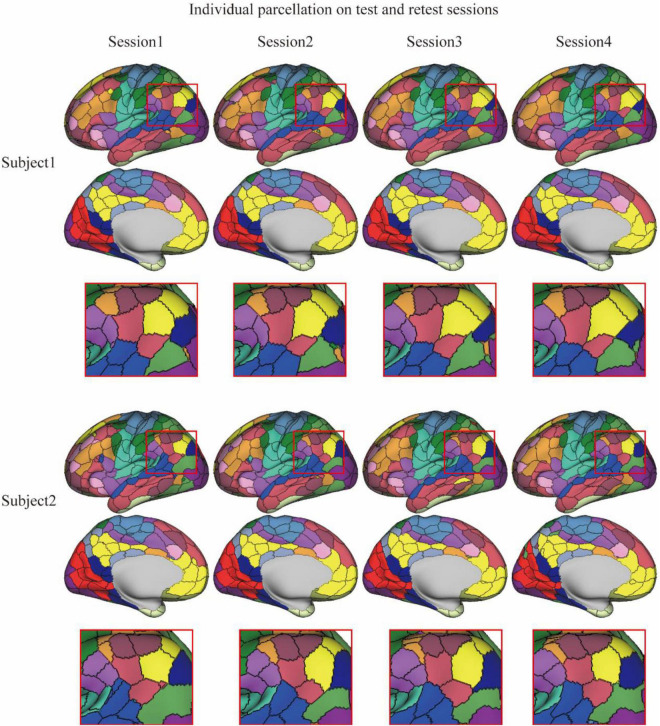
Individualized brain parcellation on two exemplar subjects and four test–retest sessions. The inferior parietal lobe (zoomed-in areas within the red rectangle) showed a similar topographic pattern across the four sessions of each subject, but detected significantly different areal topography between two subjects.

The reliability of the MSGCN parcellation was further validated on the test–retest datasets, by revealing reliable cortical parcellation on the same subject and detecting notable variations in the areal topographic organization between subjects. We also found much higher similarity in the parcellation maps among multiple sessions of the same subject compared with similarity between different subjects (dice = 0.9122 ± 0.011 and 0.8370 ± 0.012, respectively). The cortical parcellation maps on two exemplar subjects are shown in Figure 2. Individual-specific areal topography was revealed, for instance, in the inferior parietal lobe (the marked region in Figure 2), indicating a similar topographic pattern across the four test–retest sessions of the same subject but largely different areal topography between subjects. Our findings suggest that the MSGCN individualized parcellation detects reliable individual differences in the areal topography.

### Interpretability of Intersubject Variability in Brain Parcellation

**FIGURE 3:**
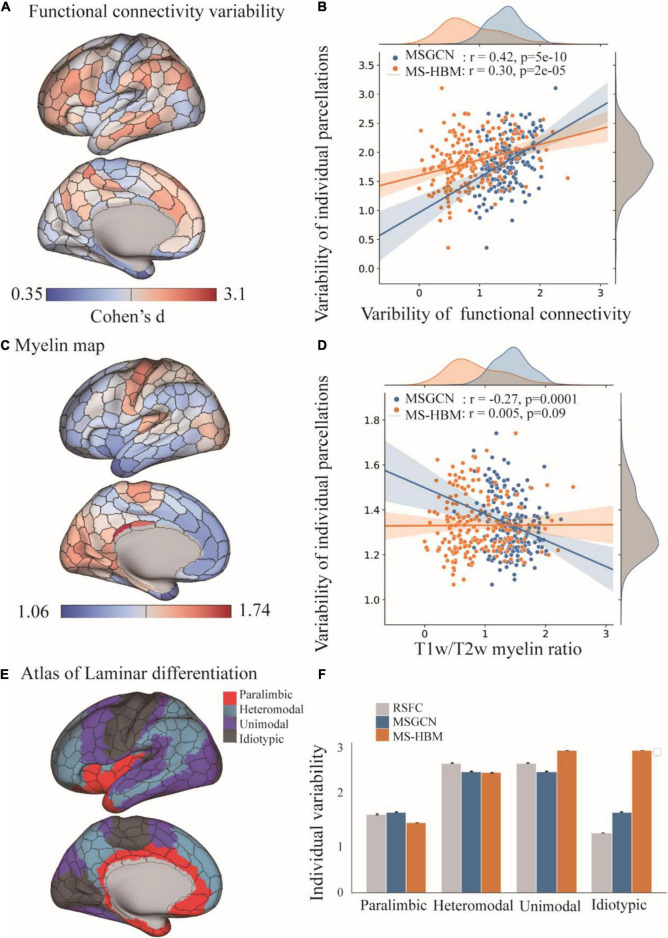
Distribution of intersubject variability in the masked semi-supervised graph convolutional neural network (MSGCN) parcellation and its association with functional, myelination, and cytoarchitecture organization. **(A)** Cortical mapping of intersubject variability in the functional connectivity profiles, with the red colors indicating high variability among subjects and blue indicating low variability. **(B)** Associations of the areal topographic variability with the variability of functional connectivity profiles. We found a strong association in the MSGCN model (*r* = 0.42, *p* < 0.001), which was much higher than multisession hierarchical Bayesian model (MS-HBM) (*r* = 0.30, *p* < 0.001). **(C)** Cortical mapping of the T1w/T2w myelin ratio map, with the red colors indicating high myelination content in the areas. **(D)** Associations of the areal topographic variability with the distribution of the myelin ratio map. We found a significant negative association in the MSGCN model (*r* = −0.27, *p* < 0.001), but not in MS-HBM (*r* = 0.005, *p* = 0.09). **(E)** Cortical mapping of laminar differentiation, with different colors representing one of the four cortical types, namely, paralimbic, heteromodal, unimodal, and idiotypic areas. **(F)** Distribution of the intersubject variability in both functional connectivity profiles and individual parcellation. The intersubject variability was evaluated by using Cohen’s *d*. Both functional connectivity profiles and MSGCN individualized parcellation showed relatively higher variability in the heteromodal and unimodal areas than the paralimbic and idiotypic areas. By contrast, the MS-HBM parcellation identified much higher variability in the idiotypic areas.

The intersubject variability of MSGCN parcellation was biologically meaningful and followed the myelination and cytoarchitectonic organizational principles of the human brain. We observed a significant association between the areal topographic variability and the variability of connectivity profiles (*r* = 0.42, *p* < 0.001, Figure 3B), both of which showed high variations in the heteromodal and unimodal regions, along with low variability in the idiotypic and paralimibic regions, regions specified by an independent atlas of laminar differentiation (Figure 3D), generally following a sensory-fugal gradient from sensory–motor and unimodal areas to heteromodal areas (Mesulam, 1998). By contrast, although exhibiting a strong association with the functional connectivity variability as well (*r* = 0.30, *p* < 0.001, Figure 3B), the MS-HBM parcellation showed a very different distribution of areal topographic variability that weakly aligned with the laminar differentiation map. For instance, much higher variability was detected in the idiotypic and unimodal regions rather than in the heteromodal regions (Figure 3D). Moreover, the areal topographic variability of the MSGCN parcellation was significantly associated with the T1w/T2w myelin ratios as well (*r* = −0.27, *p* < 0.001), indicating low variability in the primary motor and visual cortex, which are heavily myelinated, and high variability in the association cortices, which are more lightly myelinated (Glasser and Van Essen, 2011). Such association with the myelination organization was missing in the MS-HBM parcellation (*r* = 0.005, *p* = 0.09). Our results suggest that the intersubject variability revealed by the MSGCN model follows the global distribution of myelo- and cytoarchitecture, as well as the variability in functional brain organization.

### Improved Functional Homogeneity With Reduced Task Variability

**FIGURE 4:**
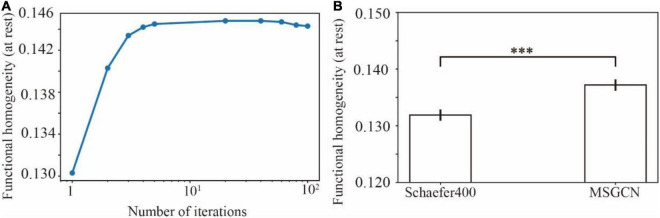
Functional homogeneity of individualized brain parcels in the masked semi-supervised graph convolutional neural network (MSGCN) parcellation. **(A)** The changes in functional homogeneity were evaluated in the validation dataset during the training processes. **(B)** Significantly higher functional homogeneity was detected in the MSGCN model (0.137 ± 0.001) than the group-registered Schaefer400 atlas (0.132 ± 0.001), as revealed by a paired *t*-test (*p* = 0.0006). ^***^p-value < 0.001.

The functional homogeneity at rest measures the internal functional consistency of brain parcels. As shown in Figure 4A, the global functional homogeneity on the validation dataset was gradually improved during the model training process. Besides, the individualized brain parcellation on the unseen test subjects also exhibited higher functional homogeneity than the initial parcellation derived from the group atlas (see Supplementary Figure 1 for an example), as detected by a paired *t*-test (*p* = 0.0006). The averaged functional homogeneity of the MSGCN parcellation was 0.137 ± 0.001 (mean ± se), evaluated on all 928 test subjects from the HCP S1200 dataset, with a 4% improvement at the whole-brain level compared with the group-registered atlas (Figure 4B).

**FIGURE 5:**
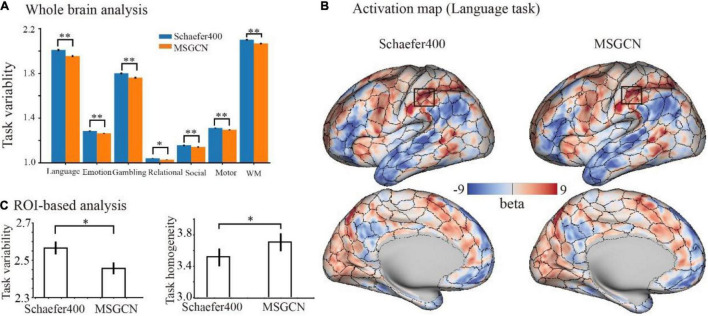
Functional alignment of the masked semi-supervised graph convolutional neural network (MSGCN) parcellation with task activations in the Human Connectome Project (HCP) tasks. **(A)** Task variability of MSGCN parcellation and Schaefer400 atlas on the seven tasks. We observed significantly lower (FDR corrected *p*-values < 0.01) regional variability in task activations by using the MSGCN parcellation (in orange) compared with the Schaefer400 atlas (in blue). **(B)** Representative activation map of the language task on a single subject, with the areal borders identified by the group-registered Schaefer400 atlas and MSGCN individualized brain parcellation, respectively. The visual assessment suggested that the fluctuations in the subject-specific task activation map went along the areal borders identified by the MSGCN parcellation but not the Schaefer400 atlas. **(C)** Task variability and homogeneity of the rectangular area marked in panel **(B)**. Quantitative comparisons suggested significantly lower variability and higher homogeneity of task activation in the detected region by using the MSGCN parcellation compared with the Schaefer400 atlas, as detected by paired *t*-tests (*p*-value = 0.02 and 0.04, respectively). *p-value < 0.05; ^**^p-value < 0.01.

On the other hand, the regional variability in task activations (task variability) measures the functional alignment between the intrinsic brain organization at rest and task-evoked brain activation during cognitive tasks. Our results showed that the MSGCN parcellation captured more homogenously distributed task activations. Overall, MSGCN parcellation showed better functional alignment at the whole-brain level for the seven tasks in HCP data, namely, language (math–story), emotion (faces–shapes), gambling (reward), relational (rel–match), social (tom–random), motor (rh-avg), and working memory (2BK-tool) tasks, with significantly reduced task variability compared with the group atlas [False Discovery Rate (FDR) corrected *p*-value < 0.05, as shown in Supplementary Table 1]. For instance, the changes in the subject-specific activation map of language task followed the areal borders identified by the MSGCN parcellation (Figure 5B), e.g., lower regional variability and higher homogeneity in the inferior parietal regions (Figure 5C). Compared with the group-registered Schaefer400 atlas, the MSGCN parcellation showed smaller variability in task activation within the detected region (*p* = 0.02) along with higher functional consistency across subjects (*p* = 0.04). Moreover, the MSGCN parcellation detected lower variability in task activations for all seven tasks at the whole-brain level (Figure 5A). Our findings indicate that the MSGCN parcellation reveals a better functional alignment across subjects in both resting state and task activation.

### Prediction Cognitive Behaviors Using Masked Semi-Supervised Graph Convolutional Neural Network Brain Parcellation

**FIGURE 6:**
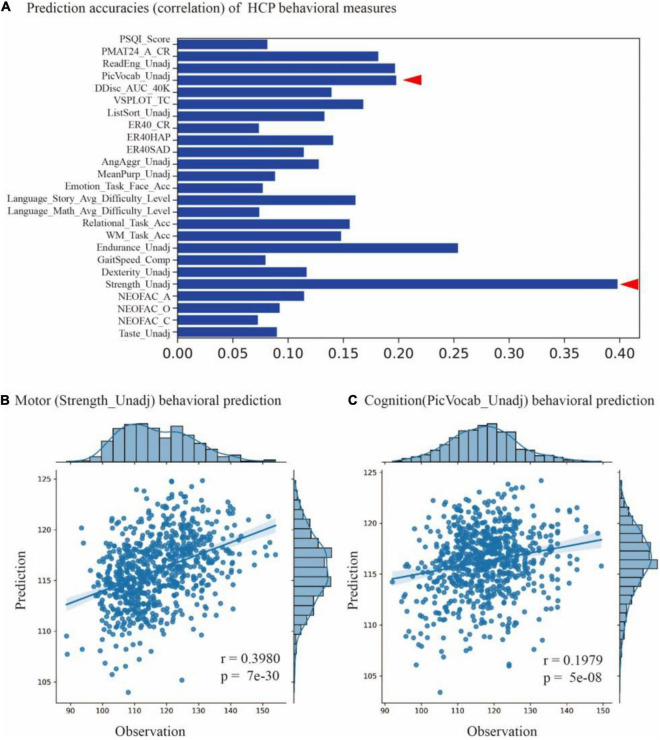
Prediction cognitive behaviors using masked semi-supervised graph convolutional neural network (MSGCN) individualized brain parcellation. **(A)** Prediction accuracies on 25 behavioral scores measured by the Pearson correlation of predicted and measured behavioral scores, with accuracy ranging from 0.07 to 0.4 (*p*-values < 0.05). **(B)** Significant associations between the predicted and measured behavioral scores for motor (Strength_Unadj, *r* = 0.398, *p* = 7e-30) and **(C)** cognition (PicVocab_Unadj, *r* = 0.1979, *p* = 5e-8), as indicated by red triangles in **(A)**.

For each of the 58 cognitive behaviors, we trained a prediction model based on the parcellation maps of 200 subjects and evaluated the model on the rest of the 728 subjects. The models achieved significant predictions (*p*-value < 0.05) on 25 behavioral scores (as shown in Figure 6A), including motor (Strength_Unadj, Endurance_Unadj), cognition (PicVocab_Unadj), language (ReadEng_Unadj), and others (see Supplementary Table 2 for a full list of 25 behavioral measures). For instance, we found significant associations between predicted and measured behavioral scores for motor (Strength_Unadj, *r* = 0.398, *p* = 7e-30) and cognition (PicVocab_Unadj, *r* = 0.1979, *p* = 5e-8), as shown in Figures 6B,C. These findings indicated that the individualized parcellation maps captured meaningful aspects of individual variability in brain topography and human cognition.

## Discussion

In this study, we propose an individualized cortical parcellation method that projects the group atlas onto individual brains by taking into account the variations in brain topography and functional connectivity. The proposed MSGCN parcellation generated highly consistent parcellation maps on multiple sessions of the same subject (intrasubject similarity = 0.89) while capturing reasonable topographic variations between subjects (intersubject similarity = 0.81). Compared with other baseline approaches including the group-registered atlas and MS-HBM, our method generated more homogeneous parcels on individual brains that strongly aligned with the intrinsic brain organization at rest and task-evoked brain activation of cognitive tasks. Moreover, the MSGCN parcellation revealed higher intersubject variability in the association cortices while keeping a stable parcellation on the primary cortex, indicating a sensory-fugal gradient from primary and unimodal areas to heteromodal areas. Such topographic variability in individualized parcellation strongly associated with the variability of functional connectivity profiles and cognitive behaviors, and generally followed the myelination, cytoarchitecture, and functional organization of the human brain.

Individualized brain parcellation has played a more and more important part in neuroscience research and clinical studies, which not only better predicted human cognition, behaviors, personality, and emotion (Kong et al., 2021), but also captured reliable and inheritable variability in the topographic organization of the human brain (Anderson et al., 2021), as well as more precise diagnosis and treatment of neurological disorders (Mueller et al., 2015; Wang et al., 2020). Yet, due to the inevitable contamination of fMRI signals by physiological noises and head motions, the traditional individualized parcellation approaches usually suffer from low generalizability on new datasets and low consistency among repeated scans. To address this issue, we applied a high-order graph convolution along with a multilayer deep learning architecture in this study. As a generalization of the conventional convolutional operations onto nongrid structures, graph convolution applies a series of low-frequency filters on the graph modes, also known as using multiple smoothing kernels on the cortical surface, and detects low fluctuations of functional connectivity along the vertex-level brain graph. The smoothing effect was controlled by the order of ChebNet graph convolution. Using this graph convolution architecture, the model generated very stable parcellations on a large population with over 75% of cortical vertices following the global topographic organization by assigning them to the same parcels among different subjects. Besides, highly consistent parcellation maps (Figures 1, 2) were generated on the multiple sessions of the same subject (dice = 0.89), along with lower consistency between subjects (dice = 0.81). Yet, high-order kernels and deep architectures may introduce over-smoothing issues in GNN, which tends to generate identical parcellation maps on all subjects. We have also observed such over-smoothing effect in our MSGCN model such that the functional homogeneity of individualized brain parcels gradually reduced when using higher-order graph convolutions (*K* > 3 in Supplementary Figure 3). Considering the tradeoff between the intersubject variability and intrasubject reliability in individualized brain parcellation, we used the third-order graph convolution along with two layers in our model, which not only revealed high consistency among test–retest sessions but also captured notable variability of brain parcellation between subjects.

The separation of intersubject variability from the randomly appearing noise components have also been considered in previous individualized parcellation models, for instance, using a hierarchical Bayesian model to quantify the variability of functional connectivity at multiple levels (Kong et al., 2021). However, by treating each area equally through a global concentration parameter on all regions, the MS-HBM approach revealed similar levels of topographic variability and heritability among the primary and association cortices (Anderson et al., 2021), which is in congruence with the well-known sensory-fugal gradient in brain organization. To avoid this effect, we chose a data-driven approach to learn the functional characteristics of each brain parcel inferred from a large group of subjects and to encode the intra- and inter-region heterogeneity of functional connectivity through a large set of graph convolutional kernels. The detected region-specific connectivity fingerprints have been proven to be highly generalizable and reliable when inferring the areal probability on unseen subjects (Figures 1, 2). The main reasons that drive this effect include (1) integrating the local connectivity context of each vertex instead of treating each vertex independently, (2) detecting the fluctuations in functional connectivity profiles within a small neighboring area in the cortical surface, and (3) indicating the areal borders on individual brains by using the gradients of function connectivity fluctuations at multiple levels. As a result, the model revealed large intersubject variability in brain parcellation, and such topographic variability was not randomly or uniformly distributed across the cerebral cortex, but rather followed the global distribution of myelination, cytoarchitecture, and functional organization of the human brain (Figure 3). The model demonstrated low variability in the primary and unimodal cortices that are heavily myelinated and large variations in the heteromodal and association cortices that are lightly myelinated. Similar associations with the myelination and cytoarchitecture organization have also been reported in both brain anatomy and function (Huntenburg et al., 2018; Demirta et al., 2019), suggesting a sensory-fugal gradient in the individual developmental and evolutionary expansion of the cerebral cortex (Glasser and Van Essen, 2011).

There are two main goals for the individualized brain parcellation, including (1) functional homogeneity of brain parcels on individual brains, and (2) consistent topographic organization across multiple sessions and different subjects. The tradeoff between these two goals was addressed by using a semi-supervised learning framework with subject-specific confidence masks. Specifically, during model training, a subject-specific confidence mask was used for the guidance of topographic alignment across subjects, indicating the true labels for a small portion of cortical vertices, i.e., the labels extracted from the group atlas (see Supplementary Figure 1 for an example). These true labels were then used to learn the association between brain topography and functional connectivity profiles and to predict the parcellation of unlabeled data in the training subjects as well as for unseen test subjects. Similar to previous approaches (Wang et al., 2015), we started from an initial parcellation (see Supplementary Figure 1 for an example) that had the highest functional homogeneity by grouping cortical vertices according to their functional connectivity profiles to ensure the functional homogeneity of brain parcels (Supplementary Figure 2). Additional modifications on this parcellation map, i.e., excluding vertices that were misaligned across sessions of the same subject or with the group atlas, introduced the important features of topographic alignment in individualized parcellation. By implementing the resulting confidence map with the semi-supervised learning, the model captured homogenous parcels on individual subjects that also followed the global topographic organization of the group atlas. It is worth noting that the MSGCN parcellation not only generated the full parcellation of half-labeled training subjects but also made predictions over unseen test subjects where no labeled data were included. The generalizability of the model barely impacts by the size of training set such that labeling all subjects from the dataset, i.e., both training and testing data were drawn from the same sets of subjects, only achieved 2% of improvement on the functional homogeneity but showed much lower intra- and intersubject reliability (Supplementary Figure 2).

The MSGCN individualized brain parcellation not only generated replicable parcels on individual brains but also captured meaningful individual variability in brain topography and human cognition. The topographic variability generally followed a sensory-fugal gradient from primary and unimodal areas to heteromodal areas, with high variations in the association cortices while keeping a stable parcellation on the primary cortex. Such topographic variability strongly associated with the variability of functional connectivity profiles, and generally followed the myelination, cytoarchitecture, and functional organization of the human brain. More importantly, the topographic variability was highly predictive to individual variability of cognitive behaviors ranging from motor to language to cognition. However, not all behavioral scores showed a strong association with the predicted scores (only 25 out of 58 behaviors with *p*-value < 0.05). This is probably due to the implementation of the Dice coefficient as the kernel function in the prediction model, which was a global measure of similarity in brain parcellation and had limited power to detect the variability in specific brain regions and networks. Other kernel functions, for instance, resting-state functional connectivity and regional morphological statistics, as well as other prediction models, could be explored in the future.

## Limitations

In the current study, we used the Schaefer cortical parcellation with 400 regions as the referenced group atlas. Yet, the proposed model is not limited to a specific atlas or specific resolutions per se, but rather easily generalized to other parcellation schemes including functional, anatomical, or multimodal atlases. It is worth noting that, in order to balance between the internal homogeneity in connectivity profiles and consistent topographic organization across subjects, the optimal setting of the graph convolution architecture should be tested when applying to new datasets and atlases, including the order of graph convolution, the number of convolutional layers, as well as the confidence masks.

## Conclusion

We proposed a masked semi-supervised GNN model for individualized brain parcellation taking into account the homogeneity of functional connectivity profiles, alignment of topographic organization across subjects, as well as the reliability of test–retest data on individual brains. Compared with other individualized approaches, the MSGCN parcellation generated more homogenous brain parcels at rest and during cognitive tasks. The model captured high topographic variability that was mainly distributed in the associated cortices while keeping a stable parcellation in the primary and unimodal areas, and generally followed the myelination, cytoarchitecture, and functional organization of the human brain. Moreover, the topographic variability strongly associated with the functional connectivity variability and significantly predicted a series of cognitive behaviors ranging from motor to language to cognition. This study provides new avenues for precise mapping of cortical areas onto individual brains, and shows potential applications in locating personalized functional areas in the diagnosis and treatment of neurological disorders.

## Data Availability Statement

The original contributions presented in the study are included in the article/Supplementary Material, further inquiries can be directed to the corresponding author.

## Ethics Statement

The studies involving human participants were reviewed and approved by the Human Connectome Project (HCP). The patients/participants provided their written informed consent to participate in this study.

## Author Contributions

YZ, WQ, and TJ: conceptualization. WQ, LM, and YZ: methodology. WQ and YZ: visualization and writing—original draft, review, and editing. WQ, YZ, TJ, and LM: investigation. All authors contributed to the article and approved the submitted version.

## Conflict of Interest

The authors declare that the research was conducted in the absence of any commercial or financial relationships that could be construed as a potential conflict of interest.

## Publisher’s Note

All claims expressed in this article are solely those of the authors and do not necessarily represent those of their affiliated organizations, or those of the publisher, the editors and the reviewers. Any product that may be evaluated in this article, or claim that may be made by its manufacturer, is not guaranteed or endorsed by the publisher.

## Footnotes

1
https://db.humanconnectome.org/data/projects/HCP_1200

2
https://github.com/Washington-University/HCPpipelines
