# Supplementary material for: Unrevealing Reliable Cortical Parcellation of Individual Brains Using Resting-State Functional Magnetic Resonance Imaging and Masked Graph Convolutions
Source: Front Neurosci. 2022 Mar 9;16:838347. doi: 10.3389/fnins.2022.838347 (PMC8959420; doi:10.3389/fnins.2022.838347)
Supplement: Supplementary file 1 [file Data_Sheet_1.pdf]

Supplementary figures and tables

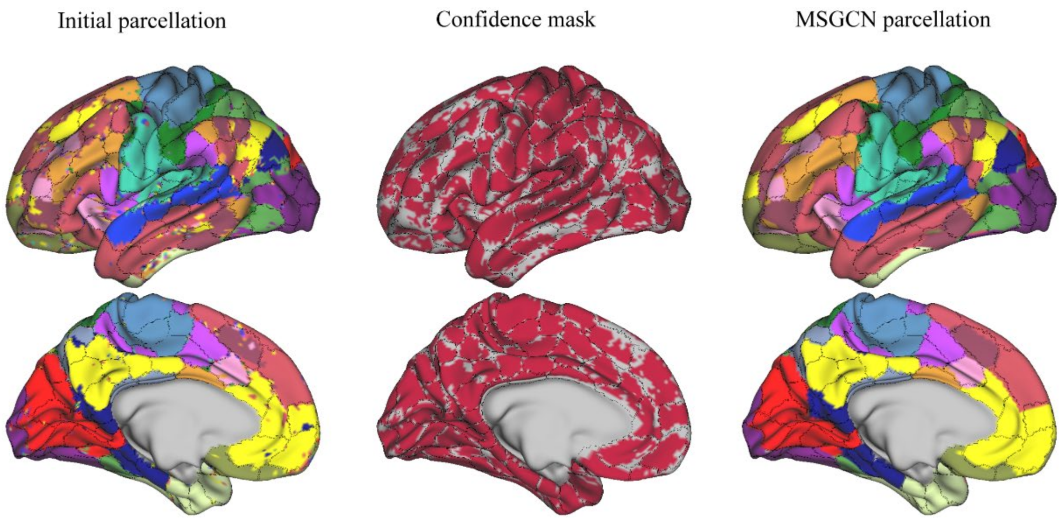

Supplement Fig.S1. Initial parcellation, confidence mask and MSGCN parcellation maps of a single subject.

Since fMRI signals are easily contaminated by various types of noises, the initial parcellation is also sensitive to small changes in functional connectivity and thus may contain isolated clusters in brain parcels. Such noisy patterns were greatly suppressed in the MSGCN parcellation by smoothing the changes of functional connectivity profiles within a small neighboring area in the cortical surface using high-order graph convolutions.

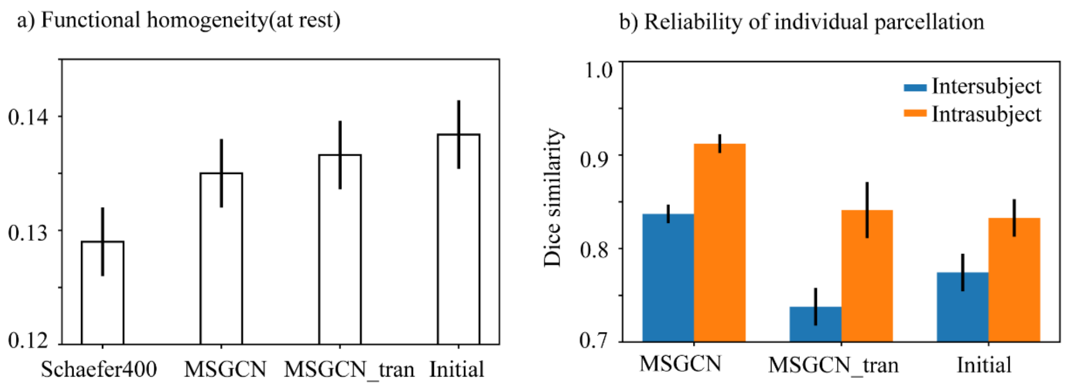

Supplement Fig.S2 Comparison of functional homogeneity and reliability of brain parcellation derived from different approaches.

a) Resting-state functional homogeneity of different brain parcellations, including the group-registered Schaefer400 atlas, MSGCN individual parcellation in an inductive way (MSGCN in the paper), MSGCN individual parcellation in a transductive way (MSGCN_tran) and initial parcellation (initial) on 44 retest subjects. Homogeneity of MSGCN_tran (0.136±0.003) is a little higher than that of MSGCN (0.135±0.003), while the initial parcellation is the largest (0.138±0.003). b) Reliability of individualized brain parcellations by using three different methods, including MSGCN (inter-Dice: 0.83±0.01, intra-Dice: 0.91±0.01), MSGCN_tran (inter-Dice: 0.73±0.02, intra-Dice: 0.84±0.03) and initial parcellation (inter-Dice: 0.77±0.02, intra-Dice: 0.83±0.02). Among them, MSGCN parcellation has the highest intra-subject similarity while initial parcellation has the lowest.

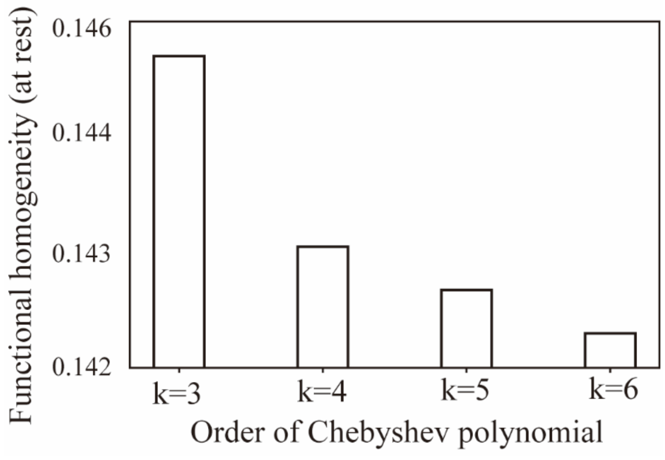

Supplement Fig.S3 Functional homogeneity of individualized brain parcellations gradually reduced when using high-order graph convolutions.

The resting-state functional homogeneity of individualized parcellations, as evaluated in the validation dataset, was gradually decreased by increasing the order of ChebNet graph convolution from 3 to 6, suggesting an over smoothing effect when using high-order graph convolutional kernels.

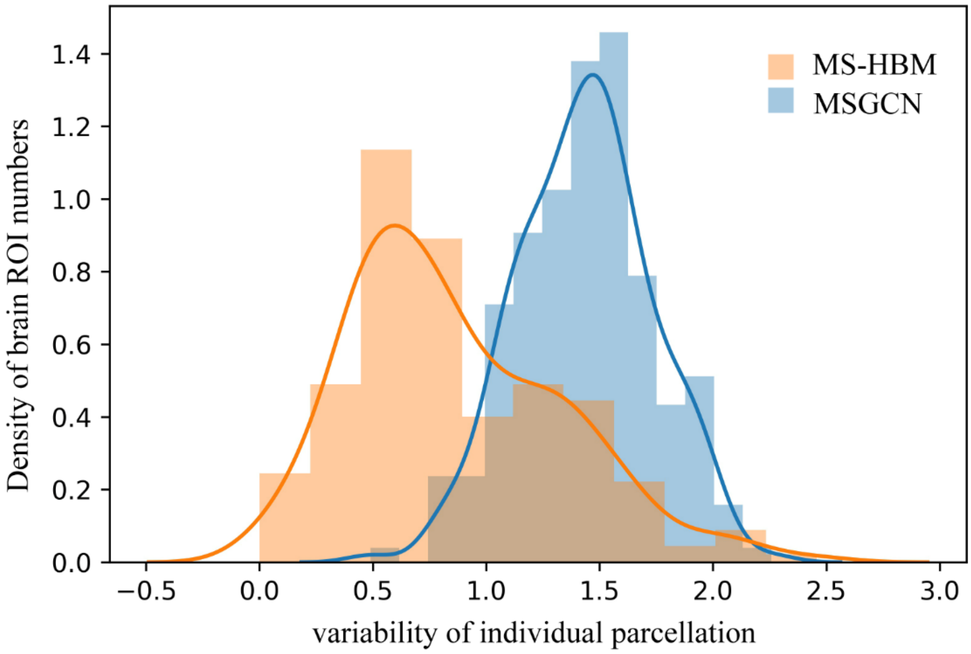

Supplement Fig.S4 Distribution of topographic variability for individualized brain parcellations using MSGCN and MS-HBM approaches.

The MSGCN captured more variations in individual parcellation maps as compared with MS-HBM (1.43±0.29 vs 0.86±0.47, paired t-test p-value<0.05).

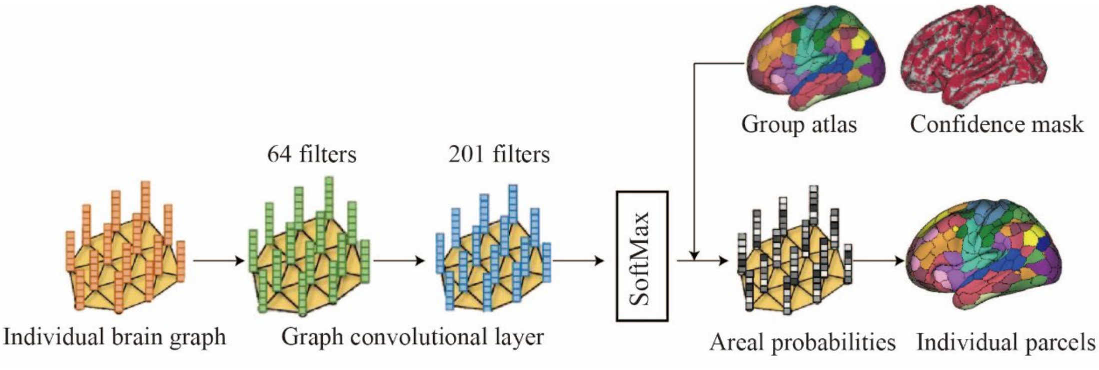

Supplement Fig.S5 Pipeline of MSGCN for individualized cortical parcellations.

The model takes the functional connectivity profiles and individual brain graph as inputs. A series of 3rd-order graph convolutions are applied to the connectivity profiles, taking into account the variations in brain topography and functional connectivity. The model includes 64 convolutional kernels in the 1st graph convolutional layer and 201 kernels in the 2nd layer. The learned graph representations of the last layer are transformed to a 201-dimensional probability vector using the SoftMax function, which is then used to infer the areal probability for each vertex.

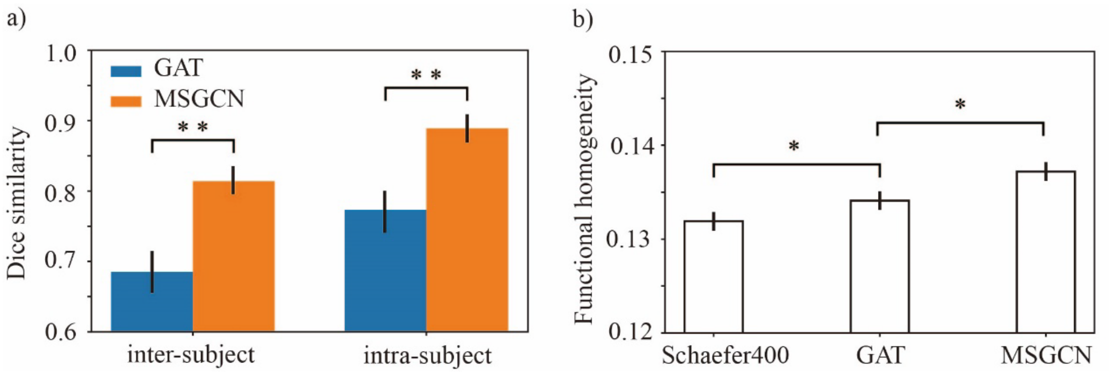

Supplement Fig.S6 MSGCN generated more reliable and functionally homogenous brain parcels than GAT.

a) The reliability of individualized brain parcellation was measured by inter- and intra-subject dice coefficients, which demonstrated more reliable parcellation schemes in the MSGCN than GAT at both between-subject (0.810±0.021 vs 0.685±0.030, paired t-test p-value<0.01) and within-subject levels (0.889±0.025 vs 0.773±0.030, paired t-test p-value<0.01). We used a two-layer GAT architecture, with 2 attention heads in the first layer and 64 convolutional kernels for each, followed by an ELU nonlinearity. A single attention head was used in the second layer with 201 convolutional kernels, followed by a softmax function to compute the areal probability of each vertex. The GAT model was trained on 50 subjects and evaluated on 928 subjects, using the same data as the current study. b) The functional homogeneity of individualized brain parcellation demonstrated more homogenous brain parcels by using MSGCN than GAT (0.137±0.001 vs 0.134±0.001, paired t-test p= 0.048). Note: * indicates p-value<0.05; ** indicates p-value<0.01.

Supplement Table S1. MSGCN parcellation showed reduced regional variability of seven HCP tasks and improved functional homogeneity at rest.

Compared to the group-registered Schaefer400 atlas, the MSGCN parcellation showed smaller variability in task activation of seven tasks, namely language (math-story), emotion (faces-shapes), gambling (reward), relational (rel-match), social (tom-random), motor (rh-avg) and working memory (2BK-tool) tasks. The MSGCN parcellation also showed higher functional homogeneity at rest. These results were still significant after corrected for multiple comparisons using FDR $p<0.05$.

	**Task variability**							**Resting-state homogeneity**
	**Language**	**Emotion**	**Social**	**Gambling**	**Motor**	**Relational**	**WM**	
p-value	1.8e-5	4.6e-4	4.9e-3	3.1e-4	9.6e-3	1.0e-2	7.7e-4	6.0e-4
FDR corrected p-value	1.4e-4	1.2e-3	6.5e-3	1.2e-3	1.1e-2	1.0e-2	1.2e-3	1.2e-3

Supplement Table S2. MSGCN parcellation significantly predicted 25 behavioral measures with p-value<0.05.

A full list of the 25 behavioral measures and their corresponding cognitive or task domains are shown in the table below.

**Cognitive domain**	**Behavioral measures**
Motor	Strength_Unadj, Endurance_Unadj, GaitSpeed_Comp, Dexterity_Unad
Cognition	PicVocab_Unadj, PMAT24_A_CR, DDisc_AUC_40K, VSPLOT_TC, ListSort_Unadj
Language	ReadEng_Unadj
Alertness	PSQI_Score
Emotion	ER40_CR, ER40HAP, ER40SAD, AngAggr_Unadj, MeanPurp_Unadj
Personality	NEOFAC_A, NEOFAC_O, NEOFAC_C
Sensory	Taste_Unadj
In-scanner task performance	Emotion_Task_Face_Acc, Language_Task_Story_Avg_Difficulty_Level, Language_Task_Math_Avg_Difficulty_Level Relational_Task_Acc WM_Task_Acc
